# Loss of trophic complexity in saline prairie lakes as indicated by stable-isotope based community-metrics

**DOI:** 10.1186/2046-9063-8-6

**Published:** 2012-03-16

**Authors:** Ryan N Cooper, Björn Wissel

**Affiliations:** 1 Department of Biology, University of Regina, 3737 Wascana Parkway, Regina, Saskatchewan S4S OA2, Canada

**Keywords:** Food web, Great Plains, Saline lakes, Stable isotopes, Trophic complexity

## Abstract

Variations in climate, watershed characteristics and lake-internal processes often result in a large variability of food-web complexity in lake ecosystems. Some of the largest ranges in these environmental parameters can be found in lakes across the northern Great Plains as they are characterized by extreme gradients in respect to lake morphometry and water chemistry, with individual parameters often varying over several orders of magnitude. To evaluate the effects of environmental conditions on trophic complexity in prairie lake food-webs, we analyzed carbon and nitrogen stable isotopes of fishes, zooplankton and littoral macroinvertebrates in 20 lakes across southern Saskatchewan. Our two-year study identified very diverse patterns of trophic complexity, with was predominantly associated with among-lake differences. Small but significant temporal effects were also detected, which were predominantly associated with changes in productivity. The most influential parameters related to changes in trophic complexity among lakes were salinity, complexity of fish assemblage, and indicators of productivity (e.g. nutrients, Chl *a*). Generally, trophic diversity, number of trophic levels, and trophic redundancy were highest in productive freshwater lakes with diverse fish communities. Surprisingly, mesosaline lakes that were characterized by very low or no predation pressure from fishes were not colonized by invertebrate predators as it is often the case in boreal systems; instead, trophic complexity was further reduced. Together, prairie lake food-webs appear to be highly sensitive to changes in salinity and the loss of piscivorous fishes, making freshwater and mesosaline lakes most vulnerable to the impacts of climate variability. This is particularly important as global circulation models predict future climate warming to have disproportionate negative impacts on hydrologic conditions in this area.

## Background

Lakes of the northern Great Plains are strongly influenced by a high variability in climate and hydrology and, as a result, are very diverse in water chemistry and lake morphometry [1,2]. Glacial retreat has left the local terrain flat and without relief [3]. Yet, 1000s of kettle lakes formed as chunks of ice broke off glaciers, forming small depressions scattered across the landscape [4]. The semi-arid to sub-humid climate in combination with the low relief resulted in the formation of closed (endorheic) drainage basins [4]. As the continuous flushing with dilute waters that is typical for boreal lakes is absent in these systems, inflows are almost exclusively associated with spring snow melt while surface water evaporation during summer is the most important loss of water [5].

Water chemistry in prairie lakes is largely a function of the flux of particulate and dissolved substances that are being delivered to the lakes during the spring snow melt, and the intensity of evaporative concentration of solutes during summer. Accordingly, lakes across the Canadian prairies show a large variability in nutrient levels, and salinity ranges from freshwater to hypersaline. Water depth includes shallow-mixed (< 3 m) systems and deep-meromictic lakes (> 20 m), and lake size ranges over several orders of magnitude [1,6]. Subsequently, the taxonomic composition of the biota in these lakes also changes with water chemistry [6]. Salinity has been reported to be most important in controlling species assemblages but other environmental factors, such as nutrient content, calcium and water depth can play crucial roles as well [7]. A recent analysis across 20 prairie lakes indicated that the strength of salinity effects differs among major taxonomic groups [8]. Accordingly, fishes were excluded with increasing salinity (> 2 g L^-1^) while littoral macroinvertebrates were ubiquitous. In contrast, zooplankton were encountered over the whole salinity range, but showed a clear transition in taxonomic composition as salinity increased. Subsequently, complexity of the fish community was associated with large changes in predation pressure on lower trophic levels, as the directional changes in prey communities (shift in size distribution) indicated that complex fish assemblages were associated with high predation pressure on invertebrates (small species dominated). As the complexity of fish community decreased, large invertebrates colonized the open water, potentially taking over the position of the top-predators in mesosaline lakes (2-12 g L^-1^). Hypersaline lakes were probably bottom-up controlled as no secondary aquatic consumers were found in these systems [8].

While food-web processes may be inferred based on presence and absence of individual taxa, this information does not provide unambiguous evidence for trophic interactions (Vander Zanden and Rasmussen1997). Accordingly, to identify how trophic dynamics and complexity may change along gradients of water chemistry, morphometry and fish assemblage, we analyzed C and N stable isotopes of individual food-web components. Carbon stable isotopes (δ^13^C) are generally used to trace the sources of primary production (energy) in food webs, while nitrogen stable isotopes (δ^15^N) indicate the trophic position of individual taxa [9,10]. Biplots of carbon and nitrogen stable isotopes are helpful to visualize the trophic relationships among taxa in individual lakes. Yet, this approach may be difficult when comparing a large number of ecosystems over broader temporal or spatial scales. The interpretation of stable isotope data becomes complicated due to system-specific differences among baselines for carbon and nitrogen isotopes that are generally associated with watershed processes rather than trophic interactions [11-13]. To address this shortcoming, we adapted recently developed multivariate methods to identify, measure, and compare the variability in food-web dynamics across a large number of systems [14,15].

Ultimately, our goals were to 1) identify trophic structure and diversity across prairie lakes using stable isotope-based community metrics, and 2) identify those environmental parameters that were most influential for changes in trophic structure. Our expectations were that hypersaline lakes would have the lowest complexity as they were inhabited only by primary producers and their consumers. Freshwater and mesosaline lakes should be characterized by similar degrees of complexity despite very different species assemblages, as the reduction in diversity due to loss of fishes should be compensated by newly immigrating invertebrate predators.

## Methods

### Study area

**Figure 1:**
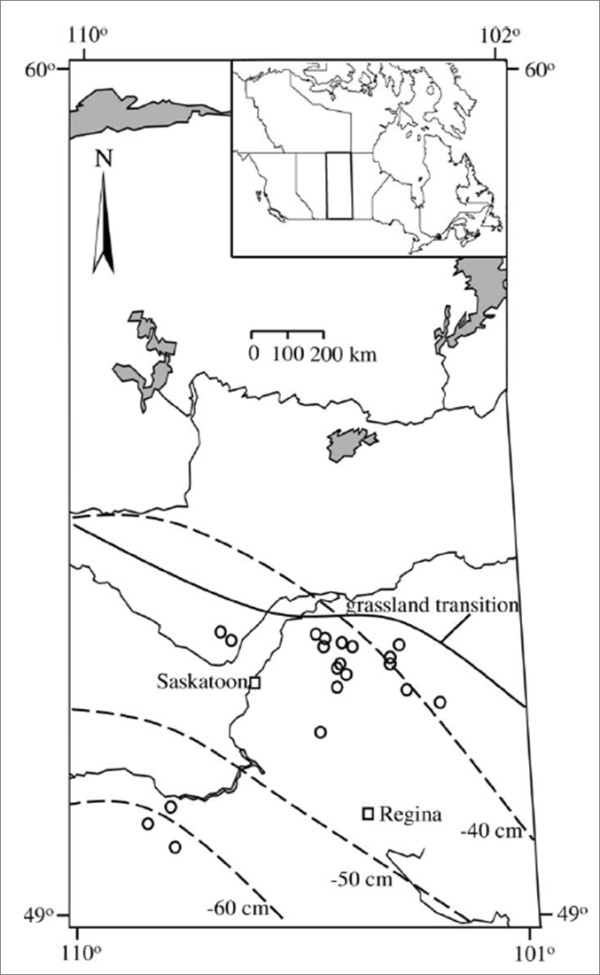
**Location of the 20 study lakes in the northern Great Plains of Saskatchewan, with sample sites shown as open circles and major cities as open squares**. Dashed lines indicate isopleths of total precipitation minus total potential evapotranspiration (cm yr^-1^) [16]. Solid line denotes grassland transition. All lakes are located within endorheic drainage basins.

Twenty lakes across southern Saskatchewan, Canada were sampled for water chemistry, fishes, zooplankton, and littoral macroinvertebrates. This prairie region (49-53°N, 103-109°W) is defined by a transition from a semi-arid climate in the southwest to a sub-humid climate in the northeast (Figure 1). Mean summer (May to September) temperatures are 13-15°C and mean precipitation during this period varies from ~118 mm in the southwest to ~240 mm in the northeast [16]. Evapotranspiration in this area is high and may exceed precipitation by 40-60 cm yr^-1 ^[16]. All study lakes were located in endorheic drainage basins with snow melt and surface run-off as main inflows and no visible (or minor seasonal) surface outflows. Maximum depths ranged from approximately 3 to 30 m and average salinity measured as total dissolved solids (TDS) ranged from fresh (< 1 g L^-1^) to hypersaline (> 50 g L^-1^). Ranges for nutrient concentrations and other water chemistry parameters were also large (Table 1). Lakes were generally polymictic, but deeper saline lakes showed meromixis with a stagnant monimolimnion.

**Table 1:** Chemical and physical characteristics of the 20 study lakes

Site	Latitude °N(dd)	Longlitude °W(dd)	Elevation (masl)	Surface Area (km^2^)	Depth (m)	TDS (g L^-1^)	Secchi (m)	Chl a (μg L^1^)	pH	TKN (μg L^1^)	NO_3_ (μg L^-1^)	NH_4_^+^ (μg L^-1^)	TP (μg L^1^)	SRP (μg L^-1^)	Ca (mg L^-1^)	DIC (mg L^-1^)	DOC (mg L^-1^)	Mixis Type
Edouard	52.38	104.33	580	1.0	5	0.4	2	25	8.3	1488	278	299	136	91	49	42	21	Polymictic
Pelletier	49.98	107.93	825	1.0	8	0.5	2	17	8.6	746	4	44	12	8	23	74	15	Polymictic
Kipabisku	52.56	104.20	522	5.2	7	0.6	2	7	8.3	1159	287	31	173	130	83	50	26	Polymictic
Lenore	52.50	104.98	537	10.0	9	1.1	2	15	8.5	809	10	45	9	2	59	59	26	Polymictic
Humboldt	52.15	105.10	544	19.1	6	1.1	1	25	8.5	1469	243	85	310	206	94	49	23	Polymictic
Clair	51.98	104.05	524	1.2	3	1	1	22	8.7	1317	221	32	33	10	73	49	25	Polymictic
Wakaw	52.66	105.58	511	10.7	7	2	2	12	8.1	1066	17	134	9	5	164	39	21	Polymictic
Shannon	52.63	105.43	549	1.0	7	2	2	12	8.8	1420	224	59	40	15	65	65	24	Polymictic
Fishing	51.83	103.50	529	32.1	12	2	3	14	8.4	1104	2	37	9	7	99	57	23	Polymictic
Rabbit	52.60	107.00	504	4.6	4	6	1	10	8.7	1734	154	30	210	153	76	99	36	Polymictic
Charron	52.40	104.33	556	4.0	7	6	2	25	8.8	2158	153	75	145	79	67	90	33	Polymictic
Arthur	52.60	107.00	504	4.6	4	6	1	10	8.7	1734	154	30	210	153	76	99	36	Polymictic
Middle	52.56	105.16	534	5.9	5	11	4	21	8.7	3191	240	77	95	6	103	95	55	Meromictic
Deadmoose	52.31	105.16	539	10.9	29	11	3	11	8.7	1788	13	113	43	9	95	65	30	Meromictic
Waldsea	52.28	105.20	533	4.7	11	12	3	10	8.6	1608	6	67	23	12	226	55	42	Meromictic
Redberry	52.71	107.15	502	60.7	11	13	3	4	8.7	2011	10	32	27	19	61	131	43	Polymictic
Antelope	50.28	108.40	701	13.8	4	15	1	14	8.9	4816	117	281	126	63	37	189	72	Polymictic
Success	50.48	108.01	715	0.7	14	20	4	9	8.9	12657	22	7585	134	115	14	350	49	Meromictic
L. Manitou	51.75	105.50	493	12.8	5	44	2	16	8.5	5741	11	133	321	47	50	91	112	Polymictic
Snakehole	50.30	108.28	858	1.3	2	64	2	7	8.5	14356	43	187	636	126	53	109	318	Polymictic

Values, except elevation and surface area, are averaged over the four sampling periods (June 2007, August 2007, June 2008, and August 2008; Rabbit Lake was not sampled in June 2008 and Middle Lake was not sampled August 2008). Lakes are sorted by increasing salinity (TDS).

### Sampling and analyses

Lakes were sampled in June and August of 2007 and 2008, with the exception of Rabbit (not sampled in June 2008) and Middle Lakes (not sampled in August 2008), which were inaccessible due to poor road conditions. Sampling for water chemistry and zooplankton was conducted at the location of maximum depth, which was generally in the central area of each lake. Dissolved oxygen (mg L^-1^), total dissolved solids (g L^-1^), water temperature (°C) and pH were measured throughout the water column in 0.5 m intervals in shallow lakes (< 5 m), or in 1 m intervals in deep lakes (> 5 m) using a YSI multi probe (model 556). Water transparency was measured with a 20 cm black and white Secchi disk. Using a tube sampler, we collected integrated, prescreened (80-μm mesh) water samples for water chemistry (total Kjeldahl nitrogen (TKN), nitrate (NO_3_^-^), total phosphorus (TP), soluble reactive phosphorus (SRP), ammonium (NH_4_^+^), dissolved inorganic carbon (DIC), dissolved organic carbon (DOC), calcium (Ca) and chlorophyll *a *concentration (Chl *a*)). The tube sampler was suspended in the water column either down to 6 m for deeper polymictic lakes, or down to 0.5 m above bottom sediments for shallower polymictic lakes, or down to 0.5 m above the monimolimnion for meromictic lakes to prevent the inclusion of water layers that are not accessible to the biota. For Chl *a*, integrated samples were filtered onto prewashed GF/C filters and stored at -10°C until extracted with an acetone: methanol: water (80:15:5 by volume) solution using standard trichromatic methods [17]. For water chemistry, samples were filtered through a 0.45 μm filter and the filtrate was stored at 4°C until analysis. Quantification of NO_3_^-^, NH_4_^+^, TKN, SRP, TDP (all μg L^-1^), and Ca (mg L^-1^) were performed at the University of Alberta Water Chemistry Laboratory following standard procedures [5,18,19]. DIC and DOC (both mg L^-1^) were analyzed on a Shimadzu TOC Analyzer 5000A at the Environmental Quality Analysis Laboratory (EQAL) at the University of Regina.

In all lakes that potentially supported fish (TDS < 20 g L^-1^), the near-shore fish community was sampled using a beach seine (2 m × 30 m, 10-mm mesh). Twice per lake, the beach seine was pulled out perpendicular to the shore, slowly brought back describing a semi-circle, and carefully retrieved to prevent escapement of fish. Fish were euthanized with buffered tricaine methonesulphonate (MS-222; [20,21]) and kept on ice until return to the laboratory, where they were frozen. Fish species were identified according to Scott and Crossman [22] and assemblages were characterized by complexity (*high complexity *- assemblages include piscivores, planktivores and benthivores; *low complexity *- assemblages lacked piscivores, and *fishless*), with complexity largely representing the number of trophic levels within the fish community.

Small and large zooplankton taxa were sampled using 80-μm (30 cm diameter) and 500-μm (50 cm diameter) conical plankton nets, respectively, which were towed vertically from maximum depth to the surface. For stable isotope analyses, samples were collected without preservation, kept on ice until the return to the laboratory where they were frozen. For species abundance and taxonomic analyses a second set of samples from each net was preserved with an ethanol-sucrose solution. Individuals were identified to species for anostraca, cladocerans, and copepods [23-25]. Rotifers were identified to genus but were not analyzed for stable isotopes due to their small size.

Littoral macroinvertebrates were collected from near-shore areas in depths up to 1 m using a large sweep net (500 μm). Samples were pooled from different littoral habitats (gravel, sand, mud, and macrophyte stands, if available) that were each sampled for approximately 10 min. Samples were preserved in ethanol to estimate presence/absence, and specimens were identified to order [24]. For stable isotope analyses, a second set of samples was collected without preservation and the samples were kept on ice until the return to the laboratory where they were frozen.

### Stable isotope analyses

Fish were thawed and a small piece (~1 g) of lateral muscle tissue [26] was extracted for stable isotope analysis [27]. The muscle tissue was rinsed with deionized water, dried at 50°C, ground to a homogeneous powder, and packed into a tin capsule. For each species per lake, 5 to 10 individuals were analyzed for stable isotopes. Zooplankton and littoral macroinvertebrates were rinsed with deionized water and abundant taxa from each group of invertebrates (cladocerans, calanoids, cyclopoids, pelagic macroinvertebrates, littoral macroinvertebrates) were selected that had sufficient sample material for stable isotope analysis. Depending on the taxon, between 1 (most littoral macroinvertebrates) and 150 individuals (small cladocerans and copepods) were packed into tin capsules and dried at 50°C.

All tin capsules were combusted in an Elemental Combustion System (Costech) that was connected to a ThermoQuest (Finnigan-MAT) Delta Plus isotope ratio mass spectrometer (IRMS). Bovine liver and wheat flour were used as internal laboratory standards. Carbon and nitrogen isotopes are reported in the conventional δ notation in ‰, relative to Vienna Pee Dee Belemnite and atmospheric N_2 _for carbon and nitrogen stable isotopes, respectively [9]. Samples that were split in the laboratory and analyzed as duplicates (n = 121) gave isotopic compositions that agreed within a 0.2‰ range for both δ^13^C and δ^15^N.

### Statistical analyses

Stable isotope studies often rely on δ^13^C and δ^15^N bi-plots to identify the importance of different energy sources (δ^13^C) and trophic positions (δ^15^N). Due to the complexity of this study (20 lakes, 2 years, 2 seasons), an additional, more synoptic approach was required to identify and quantify the changes in food-web complexity along environmental gradients.

First, to quantify the within-lake variability in stable isotope values for individual taxa, we analyzed the range of δ^13^C and δ^15^N values for common taxonomic groups within lakes over the 2-year period. We conducted this analysis on cladocerans, copepods and amphipods as these taxa were available for most sampling dates in almost all lakes. Calanoid and cyclopoid copepods were pooled for this analysis because no significant differences in temporal variability were detected between these two groups when they co-occurred in lakes (*p *> 0.05). As the range in stable isotope values differed among lakes, we conducted multiple linear regressions to identify those environmental parameters that were most influential in explaining the observed patterns. Ranges over the two-year span in δ^13^C and δ^15^N of cladocerans and copepods were independent variables, and averaged (over the four sampling events), log_10_-transformed values of environmental parameters were dependent variables.

Subsequently, to identify if the temporal variability in stable isotope values was random or followed systematic temporal shifts between seasons or years, we used Euclidian geometry and circular statistics [15]. The analysis was conducted for two different types of time intervals: 1) June 2007 to August 2007, and June 2008 to August 2008 to represent seasonal changes, and 2) June 2007 to June 2008, and August 2007 to August 2008 for annual differences. We included cladocerans, copepods, and amphipods into the analyses as these taxa were ubiquitous and occurred at all sampling times (see above). For each taxon, temporal changes in isotopic values between two δ^13^C-δ^15^N bi-plots (representing two time periods) were expressed as angle of change (θ) and magnitude of change (vector), which together characterized the directional isotopic change between the two time periods. Subsequently, from all the taxa within each lake, a mean angle of change (μ) was calculated, and Rayleigh's test was used to determine if μ was significant. A significant change with an angle around 90° or 270° indicated an increase or decrease in δ^13^C, respectively, while a respective angle around 360° or 180° represented an increase or decrease in δ^15^N. All circular statistics were calculated using Oriana 3.0.

Finally, to characterize changes in trophic structure among lakes we calculated lake-specific stable-isotope based community metrics [28]. There are six community-wide measures to determine the characteristics of food-web structure. The first four reflect trophic diversity within δ^13^C-δ^15^N bi-plots, while the other two represent trophic redundancy, or how closely positioned taxa are to each other within their respective niches. 1) Range in δ^13^C (CR), measured as difference between maximum and minimum δ^13^C values. A large CR implies a significant difference in basal resources, which demonstrates distinct niches (e.g., littoral vs. pelagic). 2) Range in δ^15^N (NR), measured as the difference between maximum and minimum δ^15^N values. This metric demonstrates the difference between the highest and lowest trophic levels, and a large NR implies more trophic levels. 3) Total area (TA), measures as convex hull area [29] enclosing all taxa within the bi-plot space. This metric represents the entire niche space or total trophic diversity. 4) Mean distance to centroid (CD), measured as average Euclidean distance of each taxa to the centroid (mean δ^13^C-δ^15^N value for all taxa in the bi-plot). This metric represents the average trophic diversity within the food web, and is not as susceptible to outliers as TA. 5) Nearest neighbor distance (NND), measured as average Euclidean distance of each taxon to its nearest neighbor within the bi-plot area. This is a measure of overall species packing, or trophic redundancy. 6) Standard deviation of nearest neighbor distance (SDNND), measuring evenness of species packing. Low values demonstrate an even distribution of taxa within the food web.

Once all metrics were calculated for the 20 study lakes (averaged over the 2-year sampling period), multiple linear regressions were used to identify environmental parameters (log_10_-transformed) that significantly contributed to the variability of these six metrics (SPSS, version 16.0). Furthermore, we conducted an Analysis of Variance (ANOVA, using SPSS, version 16.0) to test if the differences in the metrics followed the three salinity ranges where changes in biodiversity occurred: freshwater (< 2 g L^-1^, n = 9); mesosaline (2-12 g L^-1^, n = 7); and saline (> 12 g L^-1^, n = 4) [8].

## Results

### Biota

Among the 21 species of zooplankton *Daphnia pulex, D. similis, D. rosea, D. galeata mendotae, Diaphanosoma birgei*, and *Ceriodaphnia lacustris *were the most abundant cladocerans. *Leptodiaptomus sicilis *and *Diaptomus nevadensis *were abundant calanoid copepods, and *Diacyclops thomasi *was the most common cyclopoid species. The brachiopod *Artemia franciscana *dominated in hypersaline lakes. Littoral sampling resulted in 18 classes of macroinvertebrates, with amphipods (*Hyalella azteca *and *Gammarus lacustris*) and zygopterans being most abundant. Yellow perch (*Perca flavescens*), nine-spine stickleback (*Pungitius pungitius*), Brook stickleback (*Culaea inconstans*), fathead minnow (*Pimephales promelas*), northern pike (*Esox lucius*), and walleye (*Sander vitreus*) were the most common fish species.

### Stable isotope patterns

The 20 study lakes showed a broad range of lake-specific variation in stable isotope values of individual taxa over the two-year study period. Linear regressions demonstrated that the variability in δ^13^C was mostly dependent on nutrient levels and productivity measures (Table 2). For cladocerans, the most important parameters were TP, SRP, and Chl *a*, and variability in δ^13^C for copepods was related to Chl *a*, SRP, Secchi depth, and TP. Amphipod variability in δ^13^C had no significant correlation to any parameters. For δ^15^N, cladoceran values had a significant positive relationship to surface area, while Secchi depth had a significant negative effect. The δ^15^N values of copepods showed a positive correlation to Chl *a*. Amphipod variability in δ^15^N values had significant relationships with SRP, Chl a, and TP. Stepwise multiple linear regressions determined that TP and fish complexity had significant positive influences on Cladoceran δ^13^C, while Chl *a *had a positive correlation to Copepod δ^13^C (Table 3). For Cladoceran δ^15^N, Secchi depth showed a significant negative correlation and lake surface area had a significant positive relationship, while SRP had a significant positive correlation to amphipod δ^15^N (Table 3). Copepod δ^13^C and Amphipod δ^15^N were not significant correlation to any environmental parameters.

**Table 2:** Pearson correlation coefficients (*r*) between environmental parameters and temporal variation (range) of δ^13^C (C) and δ^15^N (N) for cladocerans (clad), copepods (cope) and amphipods (amph) over the two year period

	clad C	clad N	cope C	cope N	amph C	amph N
	
Variables	*r*					
Salinity	-0.33	-0.14	0.01	-0.09	0.18	0.03
SRP	**0.46**	0.19	**0.43**	-0.01	0.01	**0.54**
TP	**0.55**	0.15	**0.37**	0.04	0.05	**0.46**
Surface Area	-0.01	0.39	0.19	0.01	0.00	-0.03
Chl *a*	**0.42**	0.20	**0.59**	**0.32**	0.09	**0.49**
Secchi	-0.32	**-0.55**	**-0.40**	-0.22	-0.09	-0.39
Fish complexity	0.13	0.23	0.04	0.24	-0.17	-0.23

Bold denotes significance (*p *< 0.05)

**Table 3:** Stepwise multiple linear regression results for temporal variation (range) in δ^13^C and δ^15^N of cladocerans, copepods and amphipods as a function of environmental parameters

Metric	Standardized coefficients	*p*	*r*^2^_adj._
Cladoceran δ^13^C	0.6 TDP + 0.1 fish complexity	< 0.01	0.50
Cladoceran δ^15^N	-0.6 Secchi + 0.4 surface area	< 0.01	0.47
Copepod δ^13^C	0.6 Chl *a*	< 0.01	0.31
Amphipod δ^15^N	0.5 SRP	< 0.01	0.24

No significant regressions (*p *> 0.05) were found for copepod δ^15^N and amphipod δ^13^C

**Figure 2:**
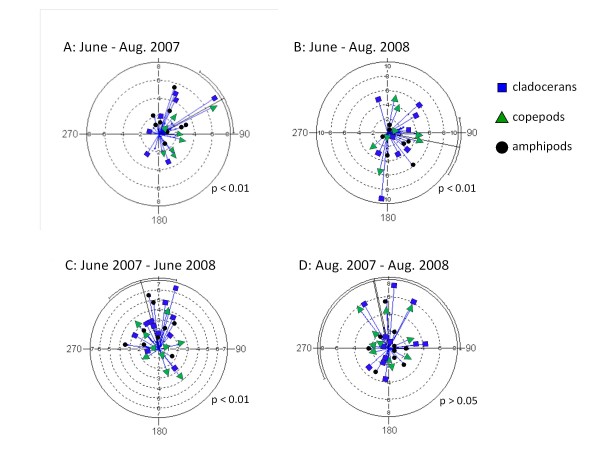
**Results of circular statistics to quantify direction and magnitude of temporal variation (Schmidt *et al.*, 2007) of δ^13^C and δ^15^N values in the 20 study lakes for selected bioata**. Periods of temporal change were either seasonal: (**A**) June to August 2007, (**B**) June to August 2008, or annual: (**C**) June 2007 to June 2008 (**D**) August 2007 to August 2008. The arrow diagrams represent the directional changes in δ^13^C and δ^15^N values between time intervals for cladocerans (blue square), copepods (green triangle), and littoral macroinvertebrates (black circle) in each individual lake. Blue arrows represent the angle of change (θ) and the length of the arrow represents the magnitude of change (vector). The mean vector of change (across all lakes and taxonomic groups, μ) is represented by the black line from the origin to the outside of the diagram. The curved line at the end of the mean vector line on the outside of the graph is the 95% confidence interval of the mean vector of change, and p-values indicate if the direction of change was significant for a particular time period. For time period (A) a significant shift was observed, which was associated with increases in both δ^13^C and δ^15^N. For time periods (B) and (C), significant shift were associated with either δ^13^C or δ^15^N, respectively, and for time period (D) no significant changes were recorded.

**Figure 3:**
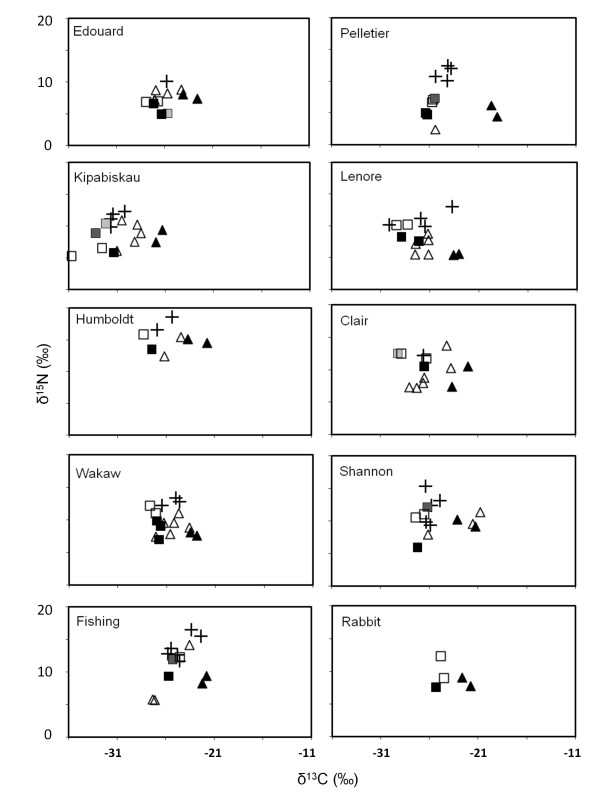
**A and B: Stable isotope bi-plots for the 20 study lakes, with δ^15^N on the Y-axis and δ^13^C on the X-axis**. Lakes are sorted by salinity (see Table 1), increasing within rows from row 1 to row 10. Data are averaged over the four sampling dates (June 2007, August 2007, June 2008, August 2008). Fish taxa are indicated by crosses (**+**), zooplankton by squares (□), and littoral invertebrate by triangles (Δ). Zooplankton is further separated into cladocerans (black squares, ■), copepods (open squares, □), invertebrate predators (i.e., *Leptodora *or *Chaoborus*; dark grey squares, ■) and other macroinvertebrates (e.g., corixids; light grey squares, ■). Within littoral invertebrates, amphipods are indicated by black triangles (▲).

Circular statistics revealed minor, yet systematic temporal changes in δ^13^C and δ^15^N. Intra-annual variation (June to August) showed a small significant shift to higher δ^13^C values for zooplankton and macroinvertebrates, indicated by mean vectors of change (μ) of 61.0° in 2007 (Rayleigh's test, *p *< 0.01, n = 36; Figure 2a) and 101.2° in 2008 (Rayleigh's test *p *< 0.01, n = 33; Figure 2b). As the angle of change was largely associated with the X-axis (δ^13^C), no systematic intra-annual changes were obvious for δ^15^N (Y-axis). Yet in both years, several lakes had lower δ^15^N during August, shown by individual angles of change (θ) associated with negative values along the Y-axis (Figure 2a and 2b). Inter-annual variation was identified only for the June sampling, which showed a small significant increase in δ^15^N from 2007 to 2008, with a mean vector of change (μ) of 345.0° (Rayleigh's test *p *< 0.01, n = 33; Figure 3).

**Figure 4:**
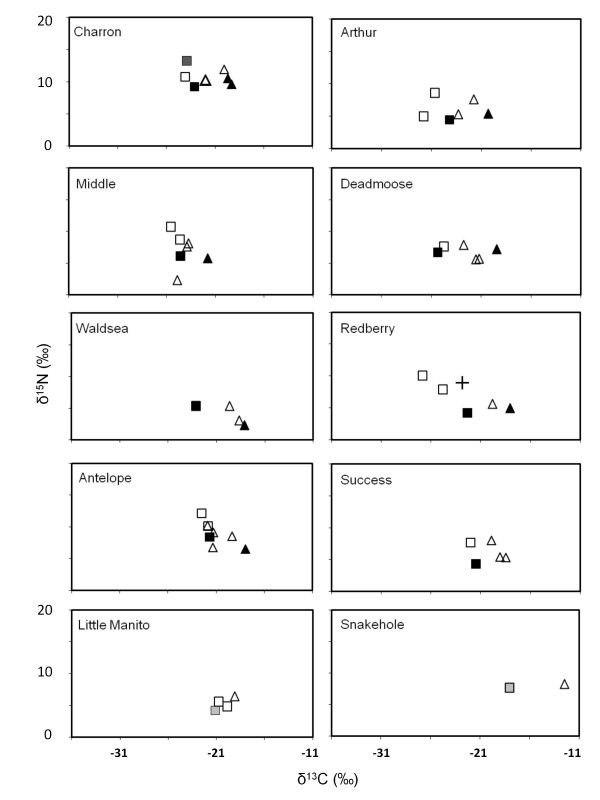
**A and B: Stable isotope bi-plots for the 20 study lakes, with δ^15^N on the Y-axis and δ^13^C on the X-axis**. Lakes are sorted by salinity (see Table 1), increasing within rows from row 1 to row 10. Data are averaged over the four sampling dates (June 2007, August 2007, June 2008, August 2008). Fish taxa are indicated by crosses (**+**), zooplankton by squares (□), and littoral invertebrate by triangles (Δ). Zooplankton is further separated into cladocerans (black squares, ■), copepods (open squares, □), invertebrate predators (i.e., *Leptodora *or *Chaoborus*; dark grey squares, ■) and other macroinvertebrates (e.g., corixids; light grey squares, ■). Within littoral invertebrates, amphipods are indicated by black triangles (▲).

*Food-web dynamics *- Based on stable isotope data, freshwater lakes had the most complex food webs, containing the greatest number of trophic levels as well as the most taxa (Figures 3 and 4). Cladocerans (*Daphnia sp*., *Ceriodaphnia sp., Bosmina sp., and Diaphanosoma sp*.) occupied the trophic position of the primary consumer within the pelagic habitat (low δ^13^C), and invertebrate predators such as *Chaoborus *and *Leptodora *were secondary pelagic consumers. Fathead minnows, as well as small (< 10 cm) walleye and yellow perch were predominantly zooplanktivorous as evidenced by intermediate δ^15^N and low δ^13^C values. In the littoral habitat (high δ^13^C), amphipods were the most common primary consumer, with predatory insect larvae (e.g. Zygoptera) as secondary consumers. Intermediate sized perch (10-15 cm), shiners, and Brook and nine-spine stickleback foraged more in the littoral zone and, at times, acted as tertiary consumers in the littoral. Larger piscivorous fish (walleye, pike, and perch > 15 cm) were top-predators in freshwater lakes and often incorporated both pelagic and littoral prey into their diet, as shown by intermediate δ^13^C and high δ^15^N values.

In contrast, saline lakes had simplified food webs and strongly reduced taxonomic richness (Figure 4). Fishes were absent in mesosaline and saline lakes, except Redberry Lake. Instead, the highest trophic levels in the pelagic and littoral areas were occupied by the predatory copepod *D. nevadensis *and zygopterans, respectively, even in Redberry Lake, where sticklebacks had slightly lower δ^15^N values than *D. nevadensis*. In hypersaline Snakehole Lake, primary consumers represented the highest trophic level in both pelagic and littoral habitats, as only halophilic *A. franciscana *and larvae of the brine fly *Ephydra sp*. were encountered. In the second hypersaline lake, Little Manitou, taxa that are usually associated with the littoral zone (e.g. harpacticoids) were isotopically indistinguishable from pelagic species such as *A. franciscana *and *L. sicilis*.

Littoral invertebrate predators (e.g. Zygoptera, Dysticidae, and corixids) did not actively invade the pelagic zone after the exclusion of fishes to take over the position of the top predator. Even though these taxa were frequently encountered in pelagic samples, based on their stable isotope values they continuously relied on littoral prey, indicating passive dispersal rather that an active migration into the open water.

Finally, in three lakes (Kipabiskau, Humboldt and Redberry), we consistently encountered copepods that had low δ^13^C values relative to all other pelagic crustaceans. These δ^13^C values were also more negative than δ^13^C of primary producers that were analyzed as particulate organic carbon (POC, data not shown).

### Stable isotope metrics

The differences in food-web structures among prairie lakes derived from bi-plots were confirmed by community-wide metrics. Freshwater lakes had a large δ^13^C range (CR; 4.9 - 9.4, mean: 6.9), compared to mesosaline (CR; 3.6 - 8.8, mean: 5.5), and saline lakes (CR: 2.0 - 5.6, mean: 3.9) (Table 4). Freshwater lakes also had the highest δ^15^N ranges (NR: 5.2 - 10.8, mean: 7.7; Table 4). Redberry Lake had the highest NR for mesosaline lakes (5.8), while Snakehole Lake had the lowest NR among all lakes (0.7). Total area (TA) showed the greatest differences between fresh and saline systems. Freshwater lakes had a TA range of 13.7 to 49.2 (mean: 34.6; Table 4). In contrast, mesosaline lakes ranged from 6.8 to 25.5 (mean: 13.6) and saline lakes ranged from 2.8 to 14.1 (mean 7.6). The mean centroid distances (CD, indicating trophic diversity) in freshwater lakes (1.9 - 3.6, mean: 2.8) were slightly larger than in mesosaline lakes (1.7 - 3.4, mean: 2.3) and saline lakes (1.1; 2.0, mean: 1.7), while saline lakes had greater mean nearest neighbor distances (NND, trophic redundancy). The standard deviations of NND (SDNND; Table 4) were not significantly different among the lakes.

**Table 4:** Summary of community-wide metrics based on δ^13^C and δ^15^N analysis [14] for the 20 study lakes

Lake Name	CR	NR	Area	CD	NND	SDNND
Eduoard	5.28	5.20	13.73	1.90	1.21	0.58
Kipabiskau	9.44	7.04	46.49	2.93	1.37	0.75
Pelletier	7.30	10.06	49.20	3.56	0.94	0.78
Lenore	7.10	7.65	38.02	3.46	1.23	0.78
Humboldt	8.46	6.17	31.34	2.77	1.67	0.63
Clair	7.19	6.53	31.45	2.79	1.20	0.88
Wakaw	4.86	6.40	21.74	2.25	1.01	0.44
Shannon	6.57	9.40	37.04	2.88	1.19	0.72
Fishing	5.57	10.77	40.23	2.88	1.11	0.72

**Mean ± SD**	**6.8 ± 1.5**	**7.7 ± 1.9**	**34 ± 11**	**2.8 ± 0.5**	**1.2 ± 0.2**	**0.70 ± 0.1**

Rabbit	3.58	4.71	11.31	1.97	1.93	0.77
Charron	4.74	4.00	12.70	2.14	1.54	0.51
Arthur	6.47	4.10	17.75	2.48	2.38	1.00
Middle	3.81	5.00	9.53	1.66	1.55	1.01
Deadmoose	5.88	2.32	6.81	2.11	1.29	0.98
Waldsea	5.03	3.01	11.36	2.22	1.94	1.23
Redberry	8.79	5.81	25.54	3.42	2.35	0.47

**Mean ± SD**	**5.5 ± 1.8**	**4.1 ± 1.2**	**13 ± 6**	**2.3 ± 0.5**	**1.9 ± 0.4**	**0.9 ± 0.3**

Antelope	4.53	5.65	14.08	1.95	1.28	0.91
Success	3.56	3.72	9.94	1.96	1.61	0.97
L. Manitou	2.02	2.22	3.36	1.06	1.40	0.30
Snakehole	5.56	0.68	2.82	1.98	2.64	0.56

**Mean ± SD**	**3.9 ± 1.5**	**3.1 ± 2.1**	**7.5 ± 5.4**	**1.7 ± 0.4**	**1.7 ± 0.6**	**0.7 ± 0.3**

Freshwater lakes are listed first, followed by mesosaline and saline lakes. A large CR (max δ^13^C - min δ^13^C) implies a significant difference in basal resources (littoral-pelagic). A large NR (max δ^15^N - min δ^15^N) demonstrates the trophic level variation. Total area (TA) encloses all species within the bi-plot space, representing total trophic diversity within the lake. Centroid distance (CD) is the average Euclidean distance of each species to the mean δ^13^C-δ^15^N value for all species in the bi-plot, measuring the average trophic diversity within the food web. Mean nearest neighbor distance (NND) is the average Euclidean distance of each species to its nearest neighbor and measures overall species packing. Standard deviation of NND (SDNND) measures evenness of species packing

Analysis of Variance (SPSS version 16.0) determined that the differences in metrics were significant for CR, NR, TA, CD, and NND (*p *< 0.05 for all) between freshwater and mesosaline lakes and freshwater and saline lakes.

Community wide metrics changed predictably with environmental variables (Table 5). CR had a positive correlation to fish complexity, salinity and TKN. NR had a significant negative association with salinity, fish complexity, TKN, DOC, and TP. Total area had a significant positive correlation to fish complexity and significant negative correlations to salinity, TKN, and DOC. Centroid distance had a significant positive correlation with fish complexity, and negative correlations with TKN, salinity, and TP. NND had positive correlations to DOC and salinity and a negative correlation to chlorophyll *a *concentration. Standard deviation of NND was not significantly correlated to any environmental parameters. Stepwise multiple linear regressions determined that salinity had a negative influence on NR, while fish complexity had significant positive correlations to CR, TA, and CD (Table 6).

**Table 5:** Pearson correlation coefficients (*r*) of the environmental influence on community-wide metrics

	CR	NR	TA	CD	NND
	
Variables	*r*				
Salinity	**-0.57**	**-0.76**	**-0.78**	**-0.62**	**0.60**
TKN	**-0.52**	**-0.70**	**-0.72**	**-0.64**	0.52
NH_4_	-0.48	-0.40	-0.49	-0.48	0.02
TP	-0.25	**-0.62**	-0.53	**-0.60**	0.47
Chl *a*	-0.22	0.19	0.04	-0.19	**-0.61**
DOC	-0.42	**-0.72**	**-0.68**	-0.56	**0.66**
Fish complexity	**0.65**	**0.74**	**0.83**	**0.72**	-0.53

Bold denotes significance. See Table 4 for abbreviations of community metrics.

**Table 6:** Results of stepwise multiple linear regression coefficients of the environmental influence on the community-wide metrics

Metric	Standardized coefficients	*p*	*r*^2^_adj._
CR	0.6 fish complexity	< 0.01	0.39
NR	-0.8 salinity - 0.6 TDP	< 0.01	0.64
TA	0.8 fish complexity	< 0.01	0.67
CD	0.7 fish complexity	< 0.01	0.50
NND	0.7 DOC - 0.6 Chl *a*	< 0.01	0.54

See Table 4 for abbreviations of community metrics. Standard deviation of NND was not significantly correlated to any of the environmental variables

## Discussion

This stable isotope study showed that lakes across the Canadian Great Plains are characterized by a very large range in trophic complexity. Trophic diversity, number of trophic levels, and trophic redundancy were highest in productive freshwater lakes with extensive fish communities. In contrast, all of these community metrics were strongly reduced in mesosaline and saline lakes. Variables that were most influential for changes in trophic complexity were salinity, fish complexity, and indicators of productivity (e.g. nutrients, Chl *a*). Furthermore, stable isotope analysis revealed that 1) lakes that were characterized by very low or no predation pressure from fishes were not colonized by larger invertebrate predators as it is often the case in boreal systems [30,31], 2) fishes could actually represent a lower trophic level than invertebrates as it was the case in Redberry Lake, and 3) energy sources other than phytoplankton that were more depleted in ^13^C might be important for pelagic communities in some lakes.

### Temporal variability

The δ^13^C values of individual zooplankton groups varied by several ‰ within individual lakes, and the degree of variability (range) was positively correlated to nutrient concentrations and algal biomass (Chl *a*). Elevated values in δ^13^C of primary producers and consumers have previously been observed in other eutrophic systems [32-34], which is associated with an increased demand for CO_2 _by photosynthetic algae and a subsequent reduction in fractionation factors during photosynthetic uptake [35]. Eutrophic systems are more often characterized by large changes in productivity [36], and the associated seasonal changes in CO_2 _demand that subsequently impact fractionation factors are likely responsible for the large observed ranges in δ^13^C. An alternative scenario is that respired CO_2 _from bottom waters with low δ^13^C [37] was taken up by phytoplankton and increased the range in δ^13^C. But this is unlikely as the study lakes and most other prairie lakes are polymictic, preventing the accumulation of respired CO_2 _in bottom waters during summer. The positive association of δ^15^N range with productivity ((Secchi (-), Chl *a *(+), nutrients (+)) is likely due to the increased importance of cyanobacteria in these eutrophic lakes. Dissolved nitrogen sources, e.g., NH_4_^+^, NO_3_^-^, and urea, have higher δ^15^N values of 5 to 8‰ in prairie lakes [38]. In contrast, atmospheric N_2 _has a δ^15^N value of 0‰ [39,40]. When dissolved nitrogen sources become limiting, the uptake of atmospheric N_2 _by cyanobacteria gains in importance, and primary producers will become isotopically more depleted. The temporary dependence on atmospheric nitrogen will result in large changes in δ^15^N values [40], which will be more pronounced and more frequently in eutrophic systems.

Circular statistics showed that temporal changes in δ^13^C of consumers followed a predictable pattern as values increased from June to August in almost all lakes in both years. In most lakes Chl *a *was highest in August, hence the trend of elevated δ^13^C during this time provides further evidence that changes in δ^13^C values were largely associated with primary production. Since there were no significant changes in δ^13^C values among years, the effect of increasing productivity over the course of the summer seems to be an annual, seasonal phenomenon in eutrophic prairie lakes. Accordingly, while the range in δ^13^C represents a measure of the differences in productivity among lakes, the seasonal changes in δ^13^C indicate an increased productivity in late summer that is common to all lakes.

In contrast to the relatively constant δ^13^C values between years, there was a small but significant shift in δ^15^N values from June 2007 to June 2008. The lower δ^15^N of primary consumers in June 2007 relative to 2008 was probably due to an increased importance of nitrogen from N_2_-fixing cyanobacteria reaching higher trophic levels during this time. Conversely, dissolved nitrogen sources with higher δ^15^N could have had larger contributions in June 2008. Humboldt Lake always had the highest δ^15^N values among all lakes (Figure 3). Past research has shown that enriched δ^15^N values are a good indicator for sewage or manure contamination [41,42]. Since such elevated δ^15^N values have been previously documented for Humboldt Lake [5,43] this contamination seems to be chronic rather that an isolated event.

### Food-web dynamics

The approach of using community-wide metrics has been applied to a number of systems since introduced by Layman et al. [28], including terrestrial [44] and aquatic ecosystems [14,45]. Despite criticism that this method is not applicable to all trophic systems [46], it is useful in trophic analysis under finite conditions, such as using trophic baselines or including co-variables, like species richness [47]. Our study applied these metrics as relative measures to evaluate ecological patterns across a large number of lakes that otherwise cannot be compared systematically. We feel that these stable-isotope based, community-wide metrics represent a more objective approach compared to previous studies on prairie or saline lake food-webs that were solely based on empirical evidence for the importance of environmental parameters. Nevertheless, we point out that the strength of our analysis is in the among-lake comparison and not in reporting absolute values of metrics for individual lakes, because due to sample-size limitations (amount) small taxa (e.g., rotifers, bosminids) were excluded from the analyses, and the pelagic fish community might be underrepresented because of the sampling design for fishes.

Most of the community-wide metrics were significantly different between food webs in freshwater (< 2 g L^-1^), mesosaline (2-12 g L^-1^), and saline lakes (> 12 g L^-1^). CR in saline lakes was probably lower due to strongly decreased species diversity in both pelagic and littoral habitats [8]. Complexity of fish assemblage had a significant positive relationship with the range in carbon stable isotope values (CR). Because fishes did not represent minimum or maximum δ^13^C values in any of the lakes, the elevated CR is likely related to increased food-web complexity when predators are present [48], and a direct effect of fishes on CR could be excluded. In three lakes (Kipabiskau, Humboldt and Redberry), we encountered copepods with lower δ^13^C values relative to all other crustaceans, indicating the presence of an additional energy source. Methane, commonly associated with low δ^13^C values [49] is unlikely to be the cause, as high sulfate concentrations in the study lakes would largely prevent methanogenesis [50]. Potentially, these low carbon stable isotope values may have been associated with sulfur bacteria [51]. Sulfate concentrations in these lakes are very high (Table 1), and strong activities of sulfur bacteria have been reported for such systems [52]. Yet, the actual mechanisms of the energy transfer from bacteria to higher trophic levels will have to be determined in future analyses.

The range in nitrogen stable isotope values (NR) was strongly associated with fish complexity. This is intuitive as lakes with a more complex fish community also encompassed more trophic levels, particularly as the increase in NR between freshwater and mesosaline lakes (3-4‰) represented approximately one trophic level. In addition to the direct effect of fish presence on diversity, predators are also known to increase food-web complexity [48]. Ultimately, the high NR values in freshwater lakes are indicative of high trophic diversity, suggesting more energy transfer to higher trophic levels [44]. Surprisingly, in mesosaline Redberry Lake, fishes did not represent the highest trophic level as the copepod *D. nevadensis *had higher δ^15^N that stickleback. As *D. nevadensis *was also associated with an additional energy source in this lake (see above), it is unclear if the high δ^15^N values were a true indicator of trophic level or if it was caused by high δ^15^N values of this additional energy source.

TA (total area) is a combination of CR and NR, as it integrates both metrics. Since TA represents total trophic diversity in food webs, it was not surprising that saline lakes had significantly lower TA values than their freshwater counterparts. CD (centroid distance), which represented average trophic diversity, was higher in freshwater lakes but not significantly different from saline lakes, possibly because the variability that is associated with differences within the littoral and pelagic areas was fairly high. Nevertheless, similar to CR, NR, and TA, regression analyses showed that CD increased with fish complexity and decreased with salinity, indicating positive and negative correlations to trophic diversity, respectively. The association of environmental parameters with nearest neighbor distance (NND, representing species packing) was opposite compared to the other metrics. NND was positively correlated to salinity and negatively correlated to fish complexity. As salinity increased, niches were occupied by fewer species, creating greater isotopic separation between individual taxa. However, the decrease of NND in freshwater lakes suggests an increase in competition and trophic redundancy [28]. Furthermore, NND was the only metric to be affected by Chl *a *(*r *= -0.61), confirming that the trophic redundancy among individual taxa was larger in eutrophic lakes.

Similar to previous community-composition analyses [8], stable isotope based metrics illustrated that salinity and fish complexity were the most significant influences within prairie lake food-webs. Salinity acted as the master variable for food-web composition in prairie lakes, having direct and indirect influences on biological (predation) and other chemical factors (e.g.: TKN and DOC) within the study lakes [7]. Changes in predation generated by the presence or absence of piscivorous and planktivorous fish created the second most important parameter regarding species composition within these ecosystems as predation influenced both pelagic and littoral communities. The fish exerted a top-down control of the food webs in both habitats [53], and with increasing salinity, their influence was removed.

Unfortunately, at this point it is not possible to clearly separate the effects of salinity and fish predation. While previous analyses focusing on community compositions in these lakes were able to statistically separate the effects of fish assemblage and salinity [8], this was not the case for regression analyses investigating trophic dynamics based on stable isotope analyses. Ongoing, experimental approaches and the inclusion of more freshwater lakes with reduced fish communities (or no fish at all) in future studies should help resolving this complex issue.

## Conclusions

Several lines of evidence indicated that salinity and complexity of fish assemblages had the most significant effects on biodiversity and food-web structure in lakes across the northern Great Plains. This is of special importance because salinity levels in prairie lakes are known to be sensitive to climatic variability [4], especially in endorheic drainage basins [6]. The IPCC (2007) reports that the Canadian prairies are one of the most at-risk areas to climate change, and small lakes that are a dominant landscape feature across the prairies will be particularly sensitive [54,55]. Species composition and biodiversity of pelagic zooplankton are most strongly affected by increasing salinities ([6-8]), and hence these taxa might undergo the most dramatic changes in the future. In contrast, lakes with a high abundance of less sensitive littoral macroinvertebrates [6,8] may become a refuge for fishes that can persist at elevated salinities and adapt to flexible foraging strategies. Furthermore, as freshwater lakes will become rarer, the controlling mechanisms of food-web composition may also switch from predation, which is typical for freshwater lakes, to competition, osmotic stress and perhaps nutrient availability (bottom-up effects) in more saline lakes.

## Competing interests

The authors declare that they have no competing interests.

## Authors' contributions

RNC and BW developed the study design and conducted the field operations. RNC prepared all samples for stable isotope analysis, performed stable- isotope based community-metrics and statistical analyses, and prepared the initial draft of the manuscript as part of a MS thesis. BW conducted all stable isotope analyses and prepared the final draft of the manuscript. All authors read and approved the final manuscript.
